# The Investigation of the Use of Prealbumin as a Tool for Nutritional Assessment in Adults Coinfected with HIV and Intestinal Helminth Parasites in KwaZulu-Natal, South Africa

**DOI:** 10.1155/2018/7805857

**Published:** 2018-07-03

**Authors:** B. T. Mkhize, M. H. L. Mabaso, S. Madurai, Z. L. Mkhize-Kwitshana

**Affiliations:** ^1^Department of Biomedical and Clinical Technology, Faculty of Health Sciences, Durban University of Technology, Durban, South Africa; ^2^Department of Medical Microbiology, School of Laboratory Medicine and Medical Sciences, College of Health Sciences, University of KwaZulu-Natal, Durban, South Africa; ^3^Epidemiology and Strategic Information Unit, HIV/AIDS, STI and TB (HAST), Human Sciences Research Council, Durban, South Africa; ^4^Global Clinical and Viral Laboratory, Durban, South Africa; ^5^Department of Biomedical Sciences, Faculty of Natural Sciences, Mangosuthu University of Technology, Durban, South Africa

## Abstract

Serum prealbumin is considered to be as important as albumin in the nutritional status assessment. However, there is relatively little evidence of its advantage over the commonly used albumin. This study investigated the use of prealbumin compared to albumin as a marker of nutritional status in adults singly and dually infected with human immunodeficiency virus (HIV) and intestinal helminths, with or without inflammatory conditions, in different body mass index (BMI) categories. This cross-sectional study was conducted in a periurban setting in KwaZulu-Natal, South Africa. Multivariate multinomial logistic regression models were fitted to investigate the effect of prealbumin and albumin in nutritional assessment among HIV and helminth individuals with or without inflammation, indicated by elevated and normal C-reactive protein (CRP) levels. In normal CRP, albumin was significantly lower in unadjusted BMI [RRR = 0.8,* p* = 0.001] and in normal weight [RRR = 0.7,* p* = 0.003] and overweight [RRR = 0.5,* p* = 0.001] participants. In elevated CRP, albumin was significantly lower [RRR = 0.8,* p* = 0.050] and prealbumin was significantly higher in unadjusted BMI [RRR = 1.2,* p* = 0.034] and overweight [RRR = 1.4,* p* = 0.052] individuals. The current study found that prealbumin can differentiate between inflammation-induced reduction of albumin and true malnutrition in adults singly or coinfected with HIV and intestinal helminths in the presence or absence of inflammation in various BMI categories.

## 1. Background

Malnutrition is a major public health problem throughout the developing regions of the world, particularly in sub-Saharan Africa [1]. In addition to the widespread malnutrition, countries in sub-Saharan Africa including South Africa (SA) carry a heavy burden of infectious diseases such as the human immunodeficiency virus (HIV) epidemic and helminth infections that coexist and are compounded by poverty [2–5]. SA has the largest proportion of individuals living with HIV globally [6]. The country also has approximately 54% of its population living in poverty [7], where conditions of malnutrition overlap with high prevalence of HIV-ascariasis and/or trichuriasis coinfections [8, 9]. The KwaZulu-Natal province, which is the epicenter of the HIV epidemic in SA, is among the poorest provinces in the country [10]. In addition, 22.7% and 15.8% of the KwaZulu-Natal population live in conditions where there is lack of adequate sanitation and safe water supplies, respectively [10], which predisposes to helminth infections [11].

While the two infections have been shown to have a deleterious effect on the immune system [12, 13], HIV and helminth infections can worsen malnutrition through various mechanisms. These include diarrhoea, disrupted intestinal mucosa lining and/or impeded absorption of nutrients, decreased nutrient intake when swallowing is painful as occurs in HIV induced candidiasis [5], and decreased appetite induced by cytokines such as tumour necrosis factor-alpha (TNF-*α*) [14, 15]. Under such conditions, malnutrition causes immune deficiency and further predisposition to infection [16], which may increase the pressure on the immune system's ability to efficiently eliminate the infectious agent. The importance of adequate nutrition on the potency of the immune system has been well established [17]. Malnutrition therefore may have an additive impact on the HIV-helminth disease progression in coinfected individuals and vice versa [4, 16, 18].

Given the complex interactions between infections, a compromised immune system, and nutritional deficiency, especially in the milieu of coexisting poverty, overweight, and obesity, reliable and objective indicators of malnutrition are essential [19], which respond to changes in nutrient intake and are not influenced by disease processes [20]. It is crucial that malnutrition is identified in obese individuals, especially in situations where an individual has nutritional deficiencies masked by obesity wherein weight, measured by body mass index (BMI), and anthropometric indices would not be reliable measures of nutritional status [21, 22]. SA has increasing prevalence of malnutrition with a predominant pattern of overweight and obesity among adults, shown as high BMI levels [23]. Obesity has been associated with malnutrition [21], which impairs immunity, further increasing the risk of infection [24]. Thus, weight gain may be misleading and inaccurate when monitoring the effectiveness of nutritional replenishment [21].

In addition to anthropometry in clinical settings, biochemical markers of serum protein levels such as total protein and albumin are commonly used to assess the nutritional status of patients, despite the fact that albumin was reported to be insensitive to acute changes in nutritional status [25], since it has a large body pool and its half-life is twenty days [20, 26]. Prealbumin has been suggested to be a more suitable biochemical marker for monitoring nutritional status, due to its short half-life of two days and its sensitivity to changes in protein-energy status within four to eight days, in both the presence and absence of inflammation [27, 28]. Prealbumin reflects more recent protein intake as opposed to albumin, which reflects long-term protein supply [29, 30]. The current study investigated the use of prealbumin as a marker of nutritional status compared to albumin in adults singly or dually infected with HIV and intestinal helminth parasites with or without inflammatory conditions as indicated by C-reactive protein (CRP) in different BMI categories in an adult population in KwaZulu-Natal, South Africa.

## 2. Methods

### 2.1. Study Setting

The study was conducted in a periurban area, randomly selected from eThekwini enumeration areas under the eThekwini Health District in the KwaZulu-Natal (KZN) province of South Africa. Currently available census data indicates that the area comprises approximately 39,000 households with approximately 30% informal settlements [31]. Poverty is widespread in this area, with low-income households, and approximately 34% of the population in the area were not economically active [31]. There is generally poor access to facilities in the area [32] with about 60% of households not having piped water inside the household [31]. The study site was a comprehensive primary healthcare clinic, providing all essential healthcare services, including HIV counselling and testing (HCT). The sociodemographic profile of the participants has been described in detail elsewhere [33].

### 2.2. Study Design and Sample Size

This study was based on a cross-sectional survey of HIV and intestinal helminths prevalence including the investigation of nutritional status, conducted between June 2014 and May 2015. A sample size of 229 adults was calculated to detect an effect size of 0.4 with 80% power and probability of 95% between the study groups.

### 2.3. Recruitment and Selection of the Study Participants

During the recruitment process, information sessions were held to inform all the clinic attendees about the study. Those willing to participate were individually given further information. After attending to any queries on the study, all those willing to participate were asked to give written informed consent before enrolment. The recruitment and enrolment process is detailed elsewhere [33]. 263 consenting adults (18 years of age and older, not on antiretroviral therapy (ART), and not pregnant if female) were enrolled in the study. Participants were then tested for HIV status for the purpose of allocating them to either a study or a reference group. Pre- and post-HIV test counselling was provided. Likewise, for classifying helminth infection status, the participants who were enrolled in the study (*n* = 263) were screened for intestinal parasites.

### 2.4. Ethical Considerations

Ethical approval to conduct the study was obtained from the University of KwaZulu-Natal Biomedical Research Committee (BREC Ref: BE 230/14). Permission to conduct the study was granted by the Provincial and eThekwini Health District office, the KwaZulu-Natal Provincial Department of Health, and the local political authorities.

The country guidelines at the time of study recruitment had set the threshold for ART initiation at 350 cells/*μ*l. Thus, HIV-infected individuals who had cluster of differentiation-4 (CD4) counts below 350 cells/*μ*l (*n* = 28) were referred to the HCT clinic and were excluded from participating in the study as per the ethical directive of protection of vulnerable individuals such as very sick or severely immunocompromised persons. Also, those who were found to be infected with intestinal parasites were referred to the clinic for anthelminthic treatment.

### 2.5. Measures

#### 2.5.1. Testing for HIV-Helminth Infection

Enrolled participants were tested for HIV status using the Alere Determine™ HIV-1/2 Ag/Ab Combo rapid test kit (Orgenics Ltd., Israel). Inconclusive results were confirmed using the Uni-Gold™ Recombigen® HIV-1/2 rapid test kit (Trinity Biotech, Ireland). Participants were also screened for intestinal helminth parasites eggs and ova. The stool samples donated by each participant on two consecutive days were screened microscopically by two trained persons, after the Kato Katz and the Mini Parasep® Faecal Parasite Concentrator (Apacor Ltd., England) preparation methods were made. The stool preparation methods are described elsewhere [33]. Blood samples were collected from each participant by a trained phlebotomist. Screened participants were allocated into four groups: (1) the uninfected/controls, (2) HIV singly infected group, (3) helminth singly infected group, and (4) HIV-helminth coinfected group. The methods of screening for HIV status and the presence of intestinal helminth parasites for allocation into these groups have been described in detail elsewhere [33].

#### 2.5.2. Anthropometry

Anthropometric measurement included weight and height, measured using a calibrated Kern® MPE scale (Kern & Sohn, Germany). The participants were weighed with light clothing and flat shoes. The scale calculated and displayed the BMI after the weight and height were keyed in. Participants were classified into the different BMI categories using World Health Organization [34] cut-off points: underweight (< 18.5), normal weight (18.5–24.9), overweight (25–29.9), and obese categories (≥ 30) for both males and females.

#### 2.5.3. Biochemical Analysis

The C-reactive protein (CRP), prealbumin, and albumin biochemical markers were analysed by a spectrophotometric auto-analyser in a South African National Accreditation System (SANAS) accredited pathology laboratory. Participants were grouped into two subgroups: (1) those showing evidence of inflammation (with elevated levels, CRP > 5 mg/l) and (2) those with no inflammation (with normal levels, CRP ≤ 5 mg/l).

### 2.6. Statistical Analysis

Descriptive statistics were used to summarize the data using frequencies and percentages for categorical data and means and standard deviations (SD) for continuous data. Four different multivariate multinomial logistic regression models were fitted to investigate the effect of prealbumin versus albumin for nutritional assessment among HIV singly infected, helminth singly infected, and HIV-helminth coinfected groups, using the uninfected group as a reference category. Model A analysed the relationship without specifying BMI category; Model B was for normal weight; Model C was for overweight; and Model D was for the obese category. Each model was fitted for individuals with no inflammation (CRP ≤ 5) and those with inflammation (CRP > 5). The effect was estimated using relative risk ratios (RRR) with 95% confidence interval (CI) significant at* p* value ≤ 0.05. Data was analysed using the statistics package STATA version 13 (College Station, Texas: Stata Corporation, USA) and SPSS version 24 (IBM Corporation, USA).

## 3. Results

### 3.1. Characteristics of the Study Participants

The mean age of the study participants was 36 years, ranging from 18 to 83 years. The majority of the participants were female (91.6%). Out of 263 participants, 40.3% were uninfected, 23.6% were infected with HIV, 23.6% were infected with helminths, and 12.5% were coinfected with HIV and helminths. Overall, the proportion of participants who had evidence of inflammation was 36.1% and the proportion of those with no inflammation was 63.9%.

**Figure 1:**
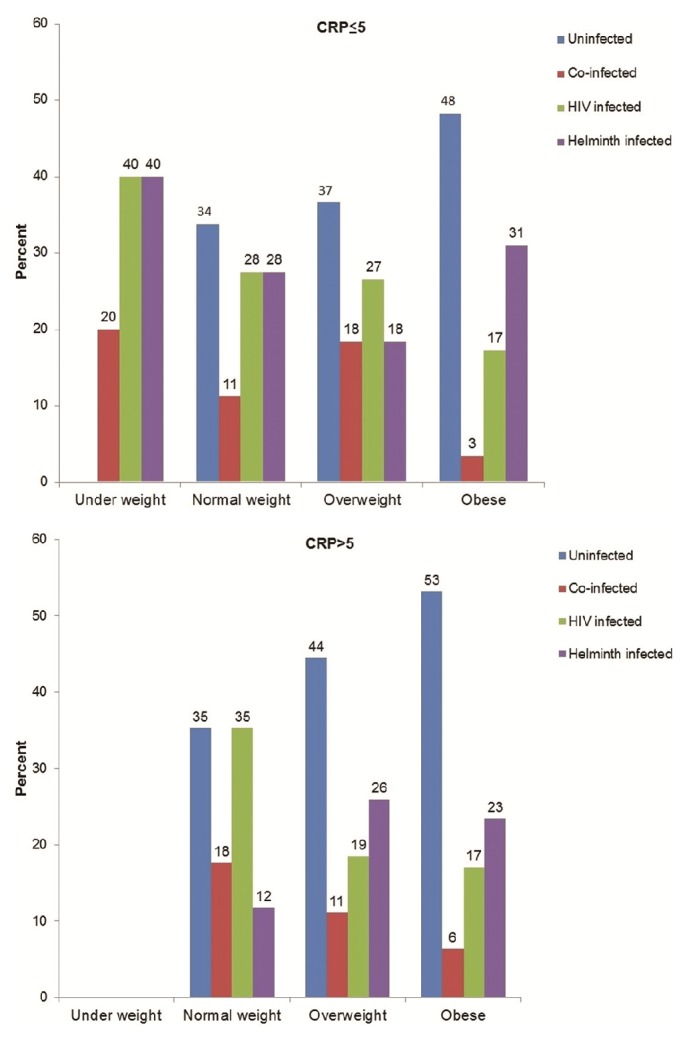
The percentage distribution of body mass index (BMI) levels among the uninfected, HIV singly infected, helminth singly infected, and HIV-helminth coinfected groups with normal (≤ 5) and high (>5) C-reactive protein (CRP) levels.

In the absence of inflammation, the majority of underweight individuals were HIV or helminth singly or dually infected, while the majority of uninfected individuals had normal weight or were overweight or obese (Figure 1). The majority of overweight and obese participants were uninfected and were mainly participants with inflammation. In the presence of inflammation (CRP > 5 mg/l), no underweight participants were recorded.

### 3.2. Biochemical Assessment of Prealbumin and Albumin Nutritional Status

**Figure 2:**
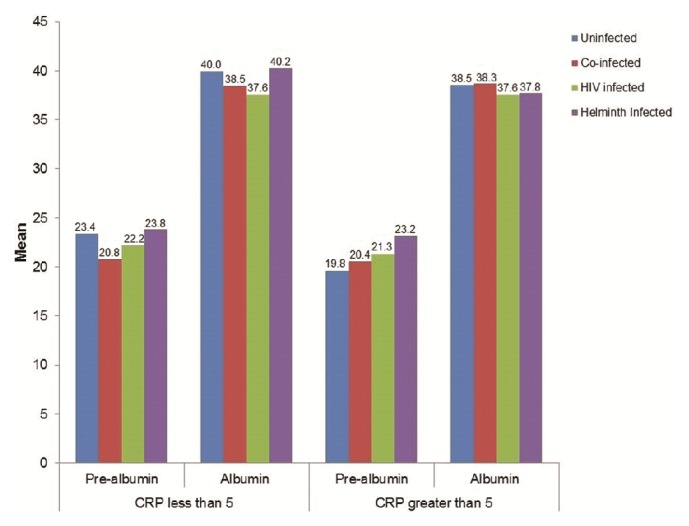
Biochemical measures of prealbumin and albumin nutritional status among the uninfected, HIV singly infected, helminth singly infected, and HIV-helminth coinfected participants with normal and high C-reactive protein (CRP) levels.

Both prealbumin and albumin were within reference ranges among all subgroups; however, both were lower in HIV singly infected and HIV-helminth coinfected groups with normal CRP levels. The mean prealbumin levels for the participants who had no inflammation (CRP ≤ 5 mg/l) were higher in the uninfected and the helminth-infected groups and lower in the HIV singly infected and the HIV-helminth coinfected groups (Figure 2). Similarly, the mean albumin levels were higher in the uninfected and helminth-infected groups and lower in the HIV-infected and HIV-helminth coinfected groups.

In participants with inflammation (CRP > 5 mg/l), the mean prealbumin levels were lower in the uninfected, HIV-infected, and HIV-helminth coinfected groups and higher in the helminth-infected group, while mean albumin was lower in the HIV-infected and helminth-infected groups and higher in the uninfected and HIV-helminth coinfected groups.

### 3.3. Models of the Effect of Prealbumin versus Albumin for Nutritional Assessment

**Figure 3:**
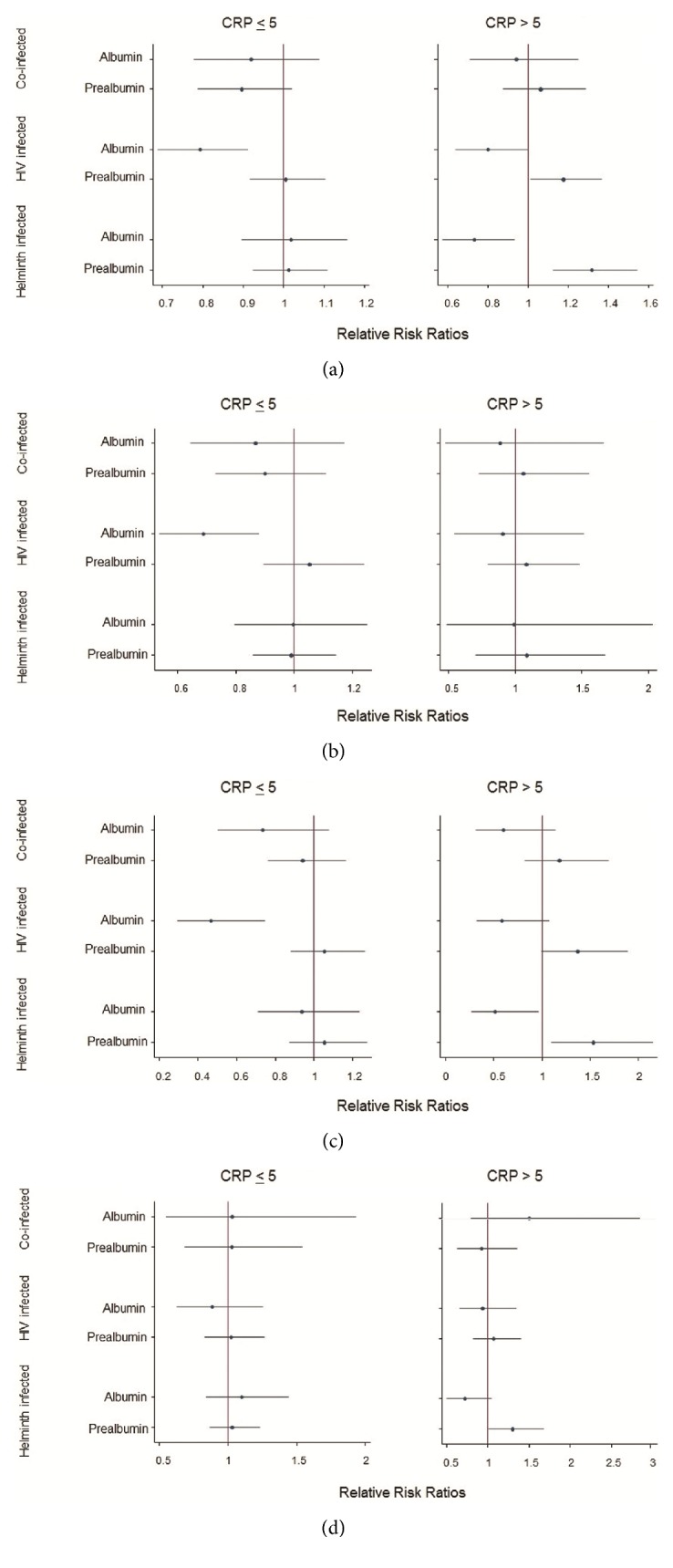
Coefficient plots for multinomial regression models of the effect of prealbumin and albumin for nutritional status assessment in (a) the body mass index (BMI) unadjusted model, (b) BMI normal weight model, (c) BMI overweight model, and (d) BMI obese model among HIV and helminth singly and dually infected groups relative to the uninfected group with C-reactive protein (CRP) levels less than 5 mg/l and greater than 5 mg/l.

The analysis revealed that HIV-helminth coinfection was associated with patterns of lower prealbumin and albumin levels across all BMI categories in the subgroup with normal CRP except in the obese, although not statistically significant. In the presence of inflammation, the pattern in the coinfected group was that of lower albumin and higher prealbumin levels in all BMI categories, except in the obese (Figure 3).

The HIV-infected group with normal CRP was associated with lower albumin and higher prealbumin across all BMI categories in both the presence and absence of inflammation, irrespective of statistical significance. Albumin was significantly lower in unadjusted BMI [RRR = 0.8 (95% CI: 0.7-0.9),* p* = 0.001] (Figure 3(a)), in normal weight [RRR = 0.7 (95% CI: 0.5-0.9),* p* = 0.003] (Figure 3(b)), and in overweight participants [RRR = 0.5 (95% CI: 0.3-0.7),* p* = 0.001] (Figure 3(c)). In the obese (Figure 3(d)), albumin was nonsignificantly lower. Prealbumin was nonsignificantly higher in all BMI categories.

In elevated CRP, albumin was significantly lower in unadjusted BMI [RRR = 0.8 (95% CI: 0.6-1.0),* p* = 0.050] and nonsignificantly lower in the normal weight, overweight, and obese. Prealbumin was significantly higher in unadjusted BMI [RRR = 1.2 (95% CI: 1.0-1.4),* p* = 0.034] and in overweight [RRR = 1.4 (95% CI: 1.0-1.9),* p* = 0.052] and nonsignificantly higher in normal weight and obese participants.

Helminth-infected group with inflammation was associated with lower albumin and higher prealbumin, irrespective of statistical significance. Albumin was significantly lower in unadjusted BMI [RRR = 0.7 (95% CI: 0.6-0.9),* p* = 0.012] and in overweight [RRR = 0.5 (95% CI: 0.3-1.0),* p* = 0.037] participants. Prealbumin was significantly higher in unadjusted BMI [RRR = 1.3 (95% CI: 1.1-1.5),* p* = 0.001], in overweight [RRR = 1.5 (95% CI: 1.1-2.1),* p* = 0.012], and in obese individuals [RRR = 1.3 (95% CI: 1.0-1.7),* p* = 0.042].

In the obese individuals, in both the presence and absence of inflammation, discordant results of both prealbumin and albumin were found in the HIV-helminth coinfected group compared to the rest of the BMI categories. The exception was in the HIV-infected group, where the pattern of lower albumin and higher prealbumin was observed in all the BMI groups, including the obese.

## 4. Discussion

In communities where single or dual infection with HIV and helminth and obesity coexist with malnutrition, it is essential that a marker that can reliably detect malnutrition in this milieu of conditions be used. The marker must ideally be unaffected by inflammation, which occurs in obesity [35], as well as in HIV and helminth infections [36, 37]. Evidence shows that obesity results in chronic low-grade inflammation, which may elevate CRP levels due to the adipose tissues releasing IL-6 and TNF-*α* and inducing the synthesis of CRP by the liver [35, 38]. Others also found an association between increased BMI and increased CRP levels, which was independent of inflammation and other factors that are known to increase CRP [39].

In the current study, despite the fact that some findings had no statistical significance, patterns were observed, which were suggestive of prealbumin being possibly useful in delineating between inflammation-induced hypoalbuminemia and true malnutrition. The key finding was that, in the absence of inflammation, participants with dual infection had lower levels of both prealbumin and albumin across all BMI categories except in the obese, which may be suggestive of malnutrition. Bishop et al. [40] and Chen et al. [41] state that low levels of both prealbumin and albumin in normal CRP are indicative of poor protein nutritional status. In the current study, it was further noted that a significant proportion of the dually infected participants with no inflammation were underweight, which is associated with malnutrition.

Furthermore, in the presence of inflammation, the pattern of lower albumin and higher prealbumin levels was observed in all the infected groups across all BMI categories, except in the coinfected participants. Similarly, this pattern was also observed in HIV-infected participants in both the absence and presence of inflammation, across all BMI categories including the obese. It is known that serum albumin levels are decreased in the presence of inflammation and infection due to the disproportionate distribution of protein between the albumin and globulin compartments [40, 42]. The assumption is that in the current study this is what caused the lower albumin in the participant groups with inflammation and/or HIV infection.

On the other hand, the higher prealbumin in these individuals may be suggestive of absence of malnutrition. Notably, the lower albumin levels in this instance may have been interpreted as indicative of malnutrition if albumin was assayed on its own and prealbumin was not included. This is a key finding that is suggestive of prealbumin being able to possibly differentiate between inflammation-induced hypoalbuminemia and true malnutrition. Saka et al. [43] found malnourished patients, with low BMI, prealbumin, and albumin levels and high CRP levels showing increased prealbumin after one week of nutritional support, which indicates prealbumin as a sensitive nutritional status marker. Others also found significantly lower prealbumin levels in patients with malnutrition compared to those who had no malnutrition; however, albumin levels were not significantly different between the two groups [44], implying that albumin was unable to distinguish between presence and absence of malnutrition, whereas prealbumin was able to indicate malnutrition.

Furthermore, in obese individuals in both the presence and absence of inflammation discordant prealbumin and albumin levels were found in the dually infected participants compared to the patterns observed in the rest of the BMI categories in the various infected groups. These study findings illustrate the difficulty in assessing malnutrition when there was dual infection and obesity in both the absence and presence of inflammation. Overweight and obesity were mostly prevalent in the participants who had evidence of inflammation, with a significant proportion mainly among the uninfected individuals. Therefore, assessing malnutrition when there is HIV and intestinal helminth coinfection and obesity requires further investigation in a longitudinal study with a large sample size.

## 5. Limitations

The cross-sectional nature of the study was limited in that it provided a “snapshot” and could not determine if malnutrition and inflammation preceded disease and changes in BMI and CRP levels. Another limitation was the small sample size, which may have resulted in the inability to determine significant associations between changes in BMI, prealbumin, and albumin in the presence and absence of inflammation in the different infections. For future studies, longitudinal cohorts with randomised sampling design and a large sample size would be recommended [45].

Furthermore, the single BMI measurements may not identify significant weight loss or gain [46]. In addition, the single CRP measurements may not accurately indicate long-term inflammation status. The fact that there was not any other measurement supporting the suggested malnutrition in the study participants was also a limitation. However, in another survey, we had observed a general low intake of micro- and macronutrients in the study participants [33].

## 6. Conclusion

The current study found prealbumin in HIV-helminth coinfection to be a possible delineator between inflammation-induced reduction and true malnutrition, since in all cases of elevated CRP it remained higher, whereas albumin was lower. To the best of our knowledge, this is the first study where CRP, prealbumin, and albumin biochemical markers were used in the investigation of malnutrition in the context of HIV-intestinal helminth coinfection in KwaZulu-Natal adults. It is recommended that CRP should essentially be included in any future investigations of malnutrition when prealbumin and albumin are used as indicators to detect malnutrition in order to delineate the inflammation-induced albumin reduction and true malnutrition in HIV and intestinal helminth singly and dually infected adults. The pattern of lower prealbumin and albumin in the coinfected group in the absence of inflammation may be suggestive of malnutrition and would require further investigation.

Furthermore, the study found that, in both the absence and presence of inflammation, obesity was associated with a discordant pattern of prealbumin and albumin which was contrary to the patterns seen in other BMI categories. This illustrates that assessing nutritional status in dual infection and obesity is a challenge, a key finding that requires further investigation in a large sample size.

Although not conclusive, these findings, however, add value to the growing research area on the investigation of the impact of HIV-helminth infection on nutritional status in South Africa, a country with increasing prevalence of obesity [23].

## Data Availability

The data underlying the findings have been included in the manuscript. If requested, the authors agree to provide copies of the original data.
